# Early versus delayed enteral nutrition in septic shock: a target trial emulation study

**DOI:** 10.3389/fnut.2026.1831524

**Published:** 2026-06-15

**Authors:** Peng Zhang, Ying Huang, Xiangcheng Zhang, Xingxing Zhu, Min Wang, Hui Xia, Tongkun Zuo

**Affiliations:** 1 Department of Critical Care Medicine, The Affiliated Huai’an No. 1 People's Hospital of Nanjing Medical University, Huai’an, China; 2 Department of Anesthesiology, The Affiliated Huaian No. 1 People’s Hospital of Nanjing Medical University, Huai’an, China

**Keywords:** causal inference, enteral nutrition, nutrition timing, septic shock, target trial emulation, vasopressor

## Abstract

**Background:**

The optimal timing of enteral nutrition (EN) initiation in septic shock remains uncertain. Although early EN is recommended for critically ill patients in current guidelines, evidence specific to septic shock is limited and inconsistent. Observational studies evaluating treatment timing are also vulnerable to immortal time bias and time-dependent confounding. Target trial emulation offers a structured approach to estimating trial-like effects from observational data while reducing these biases. We therefore emulated a target trial to assess the association between EN timing and clinical outcomes in septic shock and to explore whether this association varied with vasopressor exposure.

**Methods:**

We conducted a retrospective target trial emulation using the MIMIC-IV critical care database. Adult patients admitted to the ICU who met Sepsis-3 criteria and developed septic shock within the first 96 h after ICU admission were eligible. Two treatment strategies were defined according to the timing of EN initiation after ICU admission: early EN (0–48 h) and delayed EN (48–96 h). A clone-censor-weight (CCW) framework was used to emulate randomization. Patients were cloned at baseline and censored when their observed treatment course deviated from the assigned strategy. Stabilized inverse probability weights were estimated using pooled logistic regression models incorporating baseline characteristics and lagged time-varying clinical variables measured at 6-h intervals. The primary outcome was 28-day mortality. Weighted Cox proportional hazards models were used to estimate hazard ratios (HRs) comparing delayed versus early EN. Secondary outcomes included ventilator-free days within 28 days (VFD28). Interaction analyses evaluated whether norepinephrine exposure modified the association between EN timing and mortality.

**Results:**

A total of 4,003 patients with septic shock were identified. Within the first 96 h after ICU admission, EN was initiated in 1,301 patients, including 575 within 48 h and 726 between 48 and 96 h. In the weighted CCW analysis, delayed EN was associated with an increased risk of 28-day mortality compared with early EN (HR 1.21, 95% CI 1.15–1.27; *p* < 0.001). The estimated 28-day mortality risk was 30.0% in the early EN group and 35.0% in the delayed group. The weighted Kaplan–Meier curves separated early after ICU admission and remained apart over 28 days. Delayed EN was also associated with fewer ventilator-free days (mean difference −1.75 days, 95% CI −2.36 to −1.04). A significant interaction was observed between EN timing and norepinephrine exposure (*p* for interaction < 0.001). The excess mortality associated with delayed EN was greatest among patients receiving lower vasopressor doses and became less pronounced at higher levels of vasopressor support. Sensitivity analyses were broadly consistent with the primary findings.

**Conclusion:**

In this target trial emulation study of septic shock, initiation of enteral nutrition within 48 h was associated with lower 28-day mortality and more ventilator-free days than initiation between 48 and 96 h. The association between EN timing and survival varied according to vasopressor exposure, with the greatest apparent benefit of early EN observed among patients receiving lower levels of vasopressor support. These findings suggest that the timing of enteral nutrition in septic shock may need to be tailored to the degree of hemodynamic instability.

## Introduction

Sepsis is life-threatening organ dysfunction caused by a dysregulated host response to infection and remains a major global health burden, causing millions of deaths worldwide each year (1, 2). Septic shock represents its most severe manifestation and remains associated with considerable morbidity and mortality despite advances in modern critical care. Gastrointestinal dysfunction is common in sepsis and may contribute to loss of barrier integrity, bacterial translocation, and amplification of systemic inflammation, thereby worsening organ failure. Nutritional support is therefore considered an essential component of supportive care in critically ill patients. Enteral nutrition (EN) is generally preferred over parenteral nutrition because it helps preserve intestinal integrity, supports mucosal immune function, and may reduce infectious complications. Accordingly, current international guidelines recommend early initiation of EN in most critically ill patients when feasible (3–5).

The appropriate timing of EN in patients with septic shock, however, remains uncertain. During the early phase of shock, patients frequently require vasopressor support and may experience impaired splanchnic perfusion together with gastrointestinal dysmotility. Under these conditions, clinicians may hesitate to initiate EN because of concerns about EN intolerance or intestinal ischemia. At the same time, delaying nutritional support may result in cumulative energy deficits and prolonged catabolism. For clinicians, the timing of EN is therefore particularly difficult in patients with ongoing circulatory instability (6, 7).

Observational studies evaluating EN timing in septic shock have reported conflicting findings. Some suggested potential benefit with earlier EN, whereas others reported neutral results or raised concern in patients receiving higher vasopressor doses. Systematic review evidence has likewise highlighted substantial heterogeneity and limited certainty in the available literature (8–11).

The available evidence is also difficult to interpret because observational studies of treatment timing are especially vulnerable to methodological bias. In routine clinical practice, the decision to initiate EN evolves over time according to a patient’s clinical trajectory, including vasopressor dose, lactate levels, and overall hemodynamic stability, rather than being determined by a fixed time threshold (6, 7, 10). When treatment initiation occurs after cohort entry, conventional observational analyses that classify patients according to future treatment exposure may introduce immortal time bias. Moreover, the factors influencing the decision to start EN often change during the course of illness and are themselves associated with mortality, creating substantial time-dependent confounding. Such biases may partly account for the inconsistent conclusions reported in earlier studies. These considerations complicate the interpretation of observational evidence and highlight the importance of methods that account for delayed treatment initiation and time-varying confounding.

Target trial emulation has emerged as a practical framework for estimating causal effects from observational data while reducing time-related bias, and its use in clinical research has increased in recent years (12, 13). By specifying the protocol of a hypothetical randomized trial and reproducing its key components within observational data, this approach aligns eligibility criteria, treatment strategies, and time zero. Within this framework, the clone-censor-weight (CCW) method enables evaluation of treatment strategies involving delayed EN by cloning individuals, censoring deviations from assigned strategies, and applying inverse probability weighting to account for informative censoring (13, 14).

In this study, we emulated a target trial to evaluate the association between early versus delayed initiation of EN and clinical outcomes in patients with septic shock using the MIMIC-IV critical care database. Using a CCW analytic framework, we aimed to estimate the effect of EN timing on 28-day mortality while accounting for time-dependent treatment decisions and potential confounding. We hypothesized that earlier initiation of EN would be associated with improved outcomes compared with delayed EN.

## Methods

This study was conducted using a large, publicly accessible electronic health record database of ICU admissions at Beth Israel Deaconess Medical Center (Boston, MA, United States) from 2008 to 2019, available via PhysioNet to qualified researchers (15). The database was established under institutional review board approval from the Massachusetts Institute of Technology and Beth Israel Deaconess Medical Center (Approval No. 27252652). All data were fully deidentified, and the requirement for informed consent was waived. All investigators completed the required data use training and accessed the database in accordance with its data use agreement. The study was conducted in accordance with the Declaration of Helsinki. The study is reported in accordance with the Strengthening the Reporting of Observational Studies in Epidemiology (STROBE) statement (16).

### Eligibility criteria for the emulated trial

Adult patients (≥18 years) admitted to the ICU were screened for eligibility. We included individuals who met Sepsis-3 criteria (1) during their first ICU stay and developed septic shock within the first 96 h after ICU admission. Septic shock was operationally defined within the 6-h interval framework by norepinephrine exposure together with a recent serum lactate concentration >2 mmol/L. Detailed definitions of baseline and time-varying variables used for cohort construction are provided in Supplementary Table S5. ICU admission was considered time zero for cohort entry.

To ensure a clinically meaningful comparison of EN timing strategies, we further restricted the cohort to patients undergoing their first ICU stay, with ICU and hospital lengths of stay of at least 24 h and ICU stay not exceeding 100 days. Only the first ICU admission per hospitalization was considered. These eligibility criteria were intended to assemble a cohort of patients with septic shock in whom clinical equipoise regarding early (0–48 h) versus delayed (48–96 h) initiation of EN could reasonably be assumed during the early phase of critical illness.

### Target trial emulation

To estimate the effect of EN timing on 28-day mortality in patients with septic shock, we emulated a target trial comparing two strategies (13): initiation of EN within 0–48 h after ICU admission (early EN) versus initiation between 48 and 96 h (delayed EN).

The ICU admission was defined as time zero. All eligible patients entered the emulation at cohort entry. Follow-up during the first 96 h was structured in discrete 6-h intervals to capture time-updated exposure and clinical covariates relevant to EN initiation. A 96-h treatment assignment window was used to distinguish the early and delayed EN strategies. The definition of early enteral nutrition (0–48 h) was based on commonly used thresholds in clinical nutrition guidelines (3–5). The 96-h treatment assignment window was selected to represent a clinically plausible grace period during which delayed initiation may occur in patients with ongoing hemodynamic instability, reflecting real-world clinical practice.

To reproduce the two treatment strategies, each eligible patient was cloned at cohort entry and assigned to both strategies. Because each patient was cloned and assigned to both strategies at cohort entry, baseline covariates were identical across strategy arms prior to weighting. During the 96-h treatment assignment window, artificial censoring was applied when the observed EN initiation deviated from the assigned strategy. Specifically, patients assigned to the early EN strategy were censored if EN had not been initiated by 48 h after ICU admission. This artificial censoring ensured adherence to the assigned strategy during the treatment assignment window. Patients assigned to the delayed EN strategy were censored if EN was initiated before 48 h or if EN had not been initiated by 96 h.

The 6-h interval structure was used to update exposure status and time-varying covariates during follow-up, while eligibility was defined at cohort entry. After completion of the 96-h treatment assignment window, patients continued to be followed for mortality through 28 days without further artificial censoring related to treatment strategy. This framework reflects clinical practice, in which EN initiation depends on evolving hemodynamic status during the early phase of septic shock. This design ensured that treatment strategies were compared from a common time zero and avoided immortal time bias associated with delayed treatment initiation.

### Outcomes

The primary outcome of the emulated trial was 28-day mortality measured from ICU admission (time zero).

The secondary outcome was ventilator-free days within 28 days (VFD28). Ventilator-free days were defined as the number of days alive and free from invasive mechanical ventilation during the first 28 days after ICU admission. Patients who died within 28 days were assigned zero ventilator-free days.

Patients were followed from cohort entry until death or 28 days, whichever occurred first.

### Missing data

Physiological variables used in time-updated models were structured in discrete 6-h intervals. Time-updated covariates were defined using prespecified lagged look-back windows within the 6-h interval structure. For lactate measurements, the most recent prior value within the allowable look-back window was used, and an indicator for lactate measurement availability was included in the censoring models.

Before model fitting, we verified that all variables required for the primary weighted Cox model (time interval variables, treatment indicator, outcome indicator, cluster identifier, and stabilized weights) contained no missing values in the analytic dataset. Therefore, no additional imputation procedures were performed for the primary analysis.

### Statistical analysis

Continuous variables were summarized as mean (standard deviation) or median [interquartile range (IQR)], and categorical variables as counts and percentages. Baseline characteristics were summarized in the unweighted cohort to describe the observed population; conventional between-group comparisons were descriptive only and were not used for causal effect estimation. To address time-dependent confounding introduced by artificial censoring, stabilized inverse probability of censoring weights were estimated over the 96-h treatment assignment window using pooled logistic regression models fitted to 6-h person-period data. The full specification of numerator and denominator models used to estimate the stabilized weights is presented in Supplementary Table S6. At each interval, the probability of remaining uncensored under each assigned strategy was estimated.

The numerator model included time since ICU admission, whereas the denominator model additionally incorporated baseline covariates and lagged time-updated clinical variables measured at the preceding interval. The denominator model included baseline demographic characteristics, comorbidity burden, admission characteristics, and illness severity at ICU admission, together with lagged lactate measures, an indicator of lactate measurement availability, and lagged norepinephrine exposure variables. All time-dependent covariates were entered in lagged form to preserve temporal ordering between covariate measurement and treatment decisions. The norepinephrine exposure used for heterogeneity analyses was derived separately from the time-updated covariates used for confounder adjustment.

Stabilized weights were calculated as the ratio of predicted probabilities from the numerator and denominator models (Supplementary Table S6). Weights were truncated at prespecified thresholds to reduce the influence of extreme values. After completion of the 96-h treatment assignment window, weights were held constant for the remainder of follow-up.

The primary analysis used a weighted Cox proportional hazards model with counting-process syntax to estimate the hazard ratio (HR) for delayed versus early EN initiation on 28-day mortality. Robust standard errors clustered by stay identifier were used to account for multiple interval contributions per individual.

Weighted Kaplan–Meier curves were constructed based on the CCW analysis to visualize survival differences between treatment strategies. Hazard ratios and corresponding confidence intervals were obtained from the primary weighted Cox proportional hazards model with robust variance estimation clustered by stay identifier. Bootstrap procedures were used in sensitivity analyses to evaluate the stability of effect estimates but were not used for primary inference. All analyses were performed in R version 4.5.0.

### Sensitivity analysis

Several prespecified sensitivity analyses were conducted to evaluate the robustness of the primary findings. First, alternative definitions of the exposure window for enteral nutrition initiation were examined (0–24 h vs. 24–96 h and 0–72 h vs. 72–96 h). Second, alternative operational definitions of septic shock were evaluated based on vasopressor exposure, elevated lactate, and their combination within the first 24 h after ICU admission. Third, a complete-case analysis was performed as a sensitivity analysis to assess whether excluding observations with missing covariate information materially changed the primary result. These sensitivity analyses followed the same target trial emulation structure and used the same general modeling strategy as in the primary analysis. These alternative definitions were chosen to evaluate the robustness of the findings across different clinically relevant time thresholds.

### Data handling

Data extraction and preprocessing were performed using structured queries applied to the MIMIC-IV database. All statistical analyses were conducted in R (R Foundation for Statistical Computing, Vienna, Austria). Time-to-event analyses were implemented using counting-process Cox proportional hazards models within a discrete 6-h interval dataset constructed for the CCW procedure.

Quality control procedures included assessment of EN initiation timing distributions, artificial censoring patterns, stabilized weight diagnostics, and baseline covariate balance before and after weighting, assessed using standardized mean differences. Detailed diagnostics of artificial censoring and weight distributions are shown in Supplementary Table S3, Supplementary Table S4, and Supplementary Figure S2. To evaluate the validity of the CCW implementation, we examined the distribution of stabilized weights, applied prespecified truncation at the 99th percentile, and assessed weight stability and effective sample size as indicators of positivity. Detailed data sources for study variables in the MIMIC-IV database are summarized in Supplementary Table S7.

### Effect heterogeneity analysis

Given that vasopressor requirement reflects both shock severity and potential compromise of splanchnic perfusion, we hypothesized that the association between EN timing and mortality might vary according to early norepinephrine exposure.

Early norepinephrine exposure was summarized as the time-weighted average of lagged norepinephrine dose during the first 48 h after ICU admission, derived from 6-h interval vasopressor records. This measure provides an integrated summary of early vasopressor intensity over time. To reduce the influence of extreme values, norepinephrine exposure was winsorized at the 99th percentile and log-transformed.

Stabilized censoring weights from the primary analysis were retained for heterogeneity modeling. Effect modification was evaluated within the CCW framework using weighted Cox proportional hazards models that included an interaction term between EN timing strategy and a restricted cubic spline of early norepinephrine exposure. This specification allowed for flexible, potentially non-linear assessment of treatment heterogeneity across the observed range of vasopressor intensity.

For clinical interpretability, a complementary analysis categorized norepinephrine exposure at the 75th percentile and formally tested for interaction between EN strategy and higher versus lower vasopressor requirement. These prespecified heterogeneity analyses were exploratory and were not used to redefine treatment recommendations. For heterogeneity analyses, early norepinephrine exposure was summarized as a separate severity modifier, while lagged time-updated norepinephrine measures were retained in the censoring models to account for evolving hemodynamic status.

The specification of the hypothetical target trial and its emulation is summarized in Supplementary Table S1.

## Results

### Study population

**Figure 1:**
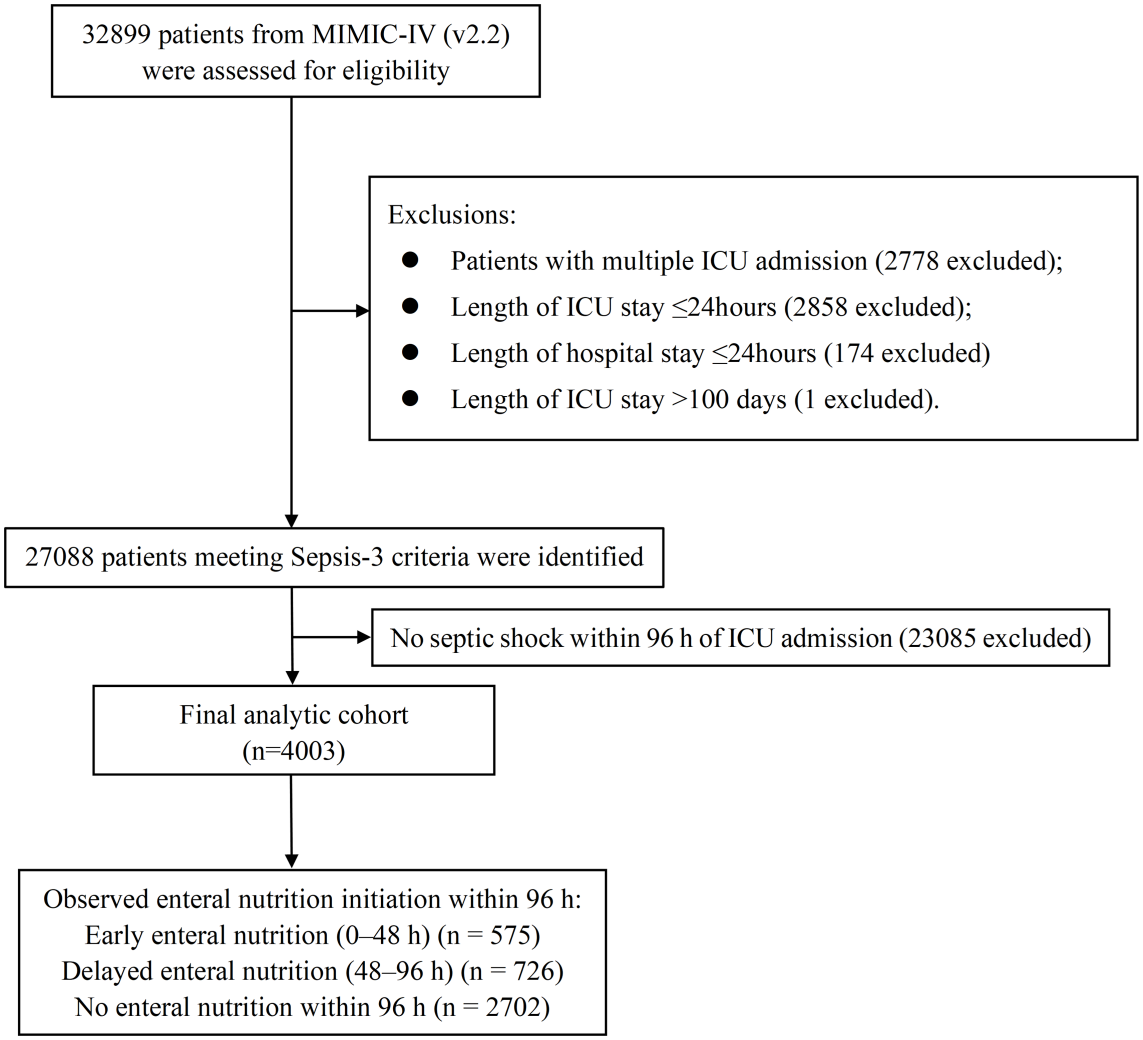
Study flowchart of cohort selection and observed timing of enteral nutrition initiation.

Among 32,899 ICU admissions recorded in MIMIC-IV, 27,088 met the Sepsis-3 definition of sepsis after application of the predefined inclusion and exclusion criteria. Among these, 23,085 did not develop septic shock within the first 96 h after ICU admission and were excluded, leaving 4,003 patients in the analytic cohort (Figure 1).

Within the first 96 h after ICU admission, 1,301 patients initiated EN, including 575 within 48 h and 726 between 48 and 96 h, whereas 2,702 patients did not initiate EN during the 96-h window. The overall timing of EN initiation is shown in Supplementary Table S2 and Supplementary Figure S1, with a median initiation time of 69.2 h (IQR 42.0–103.2). These observed initiation patterns informed adherence to the emulated early and delayed treatment strategies in the CCW analysis.

Baseline characteristics of the two groups are presented in Table 1. Patients in the early and delayed groups had broadly similar demographic profiles, comorbidity burdens, and illness severity at ICU admission. Median age was 68 years in the early group and 66 years in the delayed group, and slightly more than half of patients in both groups were male. Median APS III scores were 68 and 70, respectively, and median SOFA scores were 4 in both groups, indicating comparable illness severity at baseline.

**Table 1:** Baseline characteristics according to observed enteral nutrition initiation timing in the unweighted cohort.

	Early EN (≤48 h) (*n* = 575)	Delayed EN (>48–96 h) (*n* = 726)	SMD
Age, years, median (IQR)	68.00 [57.00, 78.00]	66.00 [54.25, 75.00]	0.175
Sex, *n* (%)			0.138
Male	315 (54.8)	447 (61.6)	
Female	260 (45.2)	279 (38.4)	
CCI, median (IQR)	6.00 [4.00, 8.00]	5.00 [3.00, 7.00]	0.101
APS III, median (IQR)	68.00 [53.00, 84.00]	70.00 [55.00, 87.00]	0.104
SOFA score, median (IQR)	4.00 [3.00, 6.00]	4.00 [3.00, 7.00]	0.088
Race, *n* (%)			0.242
White, *n* (%)	300 (52.2)	446 (61.4)	
Black, *n* (%)	93 (16.2)	65 (9.0)	
Asian, *n* (%)	21 (3.7)	25 (3.4)	
Other, *n* (%)	161 (28.0)	190 (26.2)	
Admission type, *n* (%)			0.207
Emergency, *n* (%)	348 (60.5)	440 (60.6)	
Urgent, *n* (%)	146 (25.4)	173 (23.8)	
Elective, *n* (%)	11 (1.9)	41 (5.6)	
Other, *n* (%)	70 (12.2)	72 (9.9)	
Infection site, *n* (%)			0.074
Lung, *n* (%)	315 (54.8)	391 (53.9)	
Abdominal, *n* (%)	50 (8.7)	74 (10.2)	
Genitourinary, *n* (%)	34 (5.9)	50 (6.9)	
Other, *n* (%)	53 (9.2)	59 (8.1)	
Unknown, *n* (%)	123 (21.4)	152 (20.9)	
Acute organ dysfunctions, *n* (%)			
Respiratory^a^	516 (89.7)	690 (95.0)	0.201
Kidney^b^	36 (6.3)	71 (9.8)	0.130
Liver^c^	19 (3.3)	34 (4.7)	0.070
Hematologic^d^	18 (3.1)	68 (9.4)	0.260
CRRT within 96 h, *n* (%)	97 (16.9)	157 (21.6)	0.121

SMD, standardized mean difference; IQR, interquartile range; CCI, Charlson Comorbidity Index; SOFA, Sequential Organ Failure Assessment; APS III, Acute Physiology Score III.

^a^Acute respiratory dysfunction: receipt of invasive mechanical ventilation. ^b^Acute renal dysfunction: creatinine > 1.2 mg/dL and a 50% increase from baseline. Patients with preexisting end-stage renal disease were not eligible to have acute renal dysfunction. ^c^Acute liver dysfunction: total bilirubin > 2.0 mg/dL and a 100% increase from baseline. ^d^Acute hematologic dysfunction: platelet count less than 100 cells/μl and a 50% decrease from baseline.

All variables required for the primary weighted survival analysis were complete in the final analytic dataset after preprocessing. Under the clone–censor–weight framework, artificial censoring occurred in 3,075 early-strategy clones (76.8%) and 2,632 delayed-strategy clones (65.8%), with median censoring times of 48 h and 96 h, respectively (Supplementary Table S3). These censoring patterns reflect adherence to the predefined early and delayed treatment strategies during the 96-h treatment assignment window. Stabilized weights showed a stable distribution, with a mean of 1.040, median 1.000, 95th percentile 1.229, 99th percentile 2.099, and maximum 19.934. As prespecified, weights were truncated at the 99th percentile to limit the influence of extreme values. The effective sample size ratio was 0.823, suggesting stable weighting and no evidence of severe positivity violations. These diagnostics are summarized in Supplementary Table S4 and Supplementary Figure S2. As expected under the cloning design, baseline covariates were exactly balanced between strategy arms at cohort entry, with standardized mean differences of zero. After weighting, standardized mean differences remained close to zero, indicating that the weighting procedure did not introduce imbalance (Supplementary Table S8).

### Primary outcome: 28-day mortality

**Figure 2:**
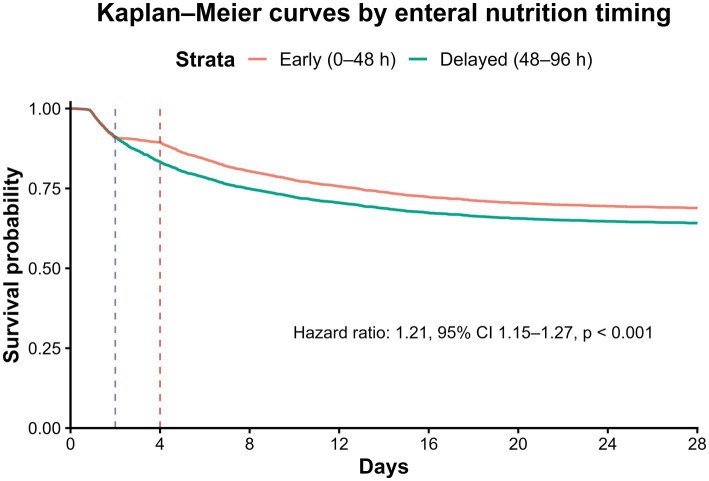
Weighted Kaplan–Meier curves comparing early and delayed enteral nutrition strategies. After clone–censor–weight (CCW) estimation, the weighted population represented patients assigned to either early enteral nutrition (0–48 h) or delayed enteral nutrition (48–96 h). Survival curves estimated from the weighted Cox model showed that early enteral nutrition was associated with a lower hazard of death within 28 days. Dashed vertical lines indicate the boundaries of the exposure windows (48 h and 96 h after ICU admission).

In the weighted Cox proportional hazards model, the hazard ratio for delayed versus early EN was 1.21 (95% CI 1.15–1.27; *p* < 0.001). The weighted Kaplan–Meier curves illustrate this difference in survival (Figure 2), with separation between the curves appearing early after ICU admission and persisting throughout the 28-day follow-up period. The estimated 28-day mortality risk was 30.0% in the early EN group and 35.0% in the delayed group, corresponding to an absolute risk difference of 5.0% (Table 2).

**Table 2:** Primary and secondary outcomes in the target trial emulation.

Outcomes	Early EN (≤48 h)	Delayed EN (>48–96 h)	Effect estimate
28-day mortality risk	30.0%	35.0%	RD 5.0%
28-day mortality			HR 1.21 (95% CI 1.15 to 1.27), *p* < 0.001
Ventilator-free days at day 28	17.81	16.06	MD −1.75 days (95% CI −2.36 to −1.04)

RD, risk difference; HR, hazard ratio; MD, mean difference. RD and MD were calculated as delayed minus early enteral nutrition.

### Secondary outcome: ventilator-free days

**Figure 3:**
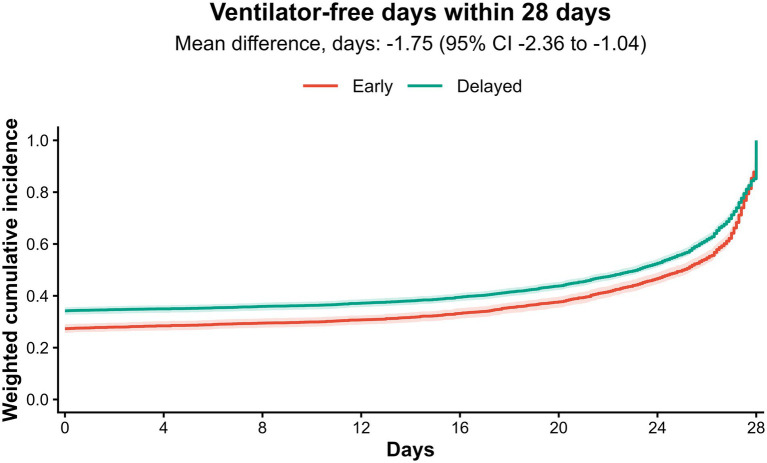
Weighted comparison of ventilator-free days within 28 days between early and delayed enteral nutrition strategies. Shaded areas represent 95% confidence intervals estimated by bootstrap resampling.

Delayed EN was also associated with fewer ventilator-free days (Figure 3 and Table 2). The weighted mean number of ventilator-free days was 17.81 days in the early group and 16.06 days in the delayed group, corresponding to a mean difference of −1.75 days (95% CI −2.36 to −1.04; delayed minus early).

### Subgroup analyses

**Figure 4:**
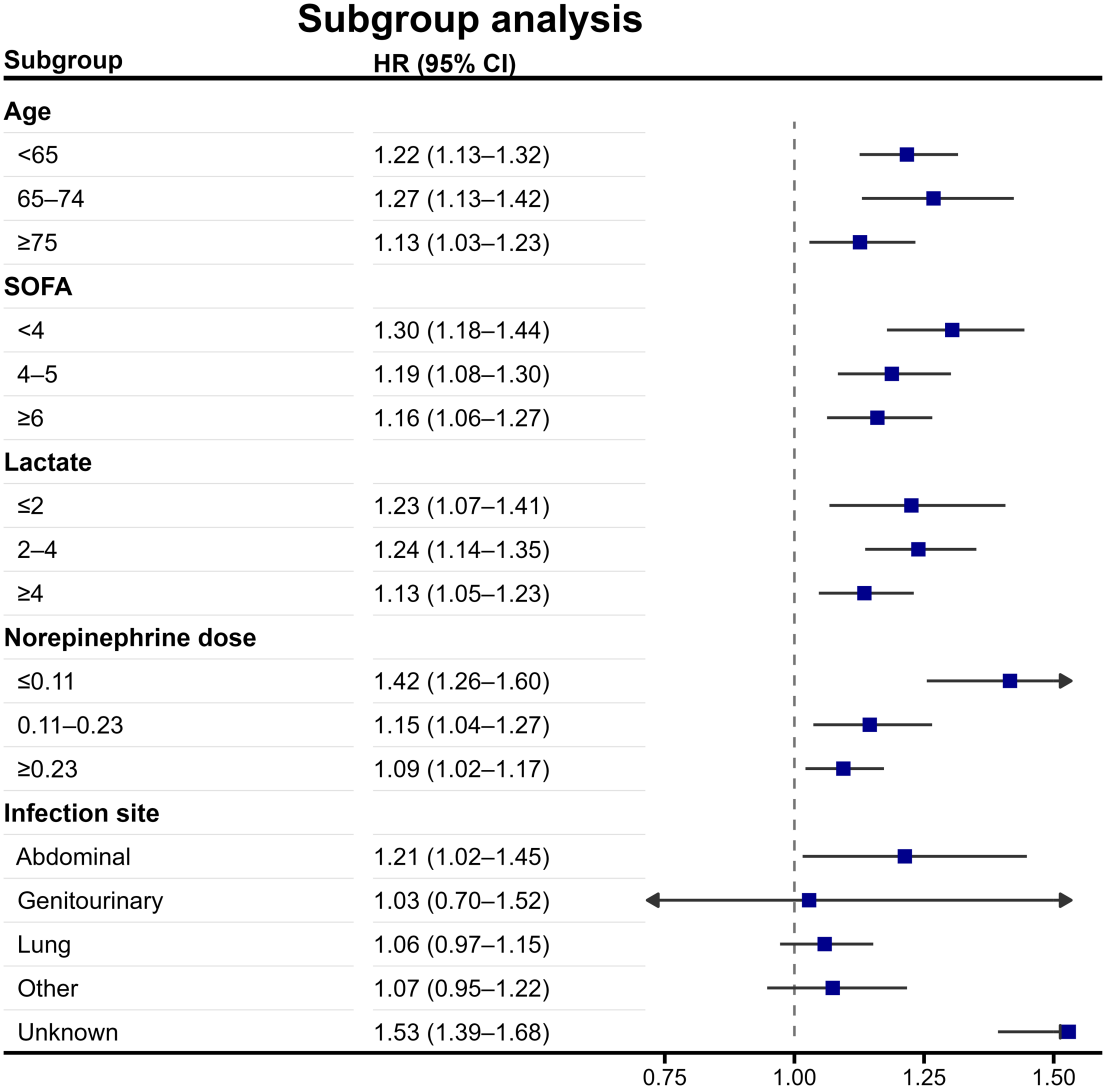
Subgroup analysis of the association between timing of enteral nutrition and 28-day mortality. Forest plot showing hazard ratios (HRs) and 95% confidence intervals (CIs) for delayed versus early enteral nutrition across predefined subgroups.

Exploratory subgroup analyses showed that the association between delayed EN and mortality was generally consistent across prespecified strata defined by age, baseline SOFA score, lactate level, norepinephrine exposure, and infection site (Figure 4). However, the magnitude of the association varied according to norepinephrine exposure.

Among patients with lower norepinephrine exposure (time-weighted average ≤0.11 μg/kg/min), delayed EN was associated with a substantially higher risk of mortality (HR 1.42, 95% CI 1.26–1.60). In contrast, among patients with higher norepinephrine exposure, the difference between early and delayed strategies appeared attenuated.

### Continuous interaction analysis

**Figure 5:**
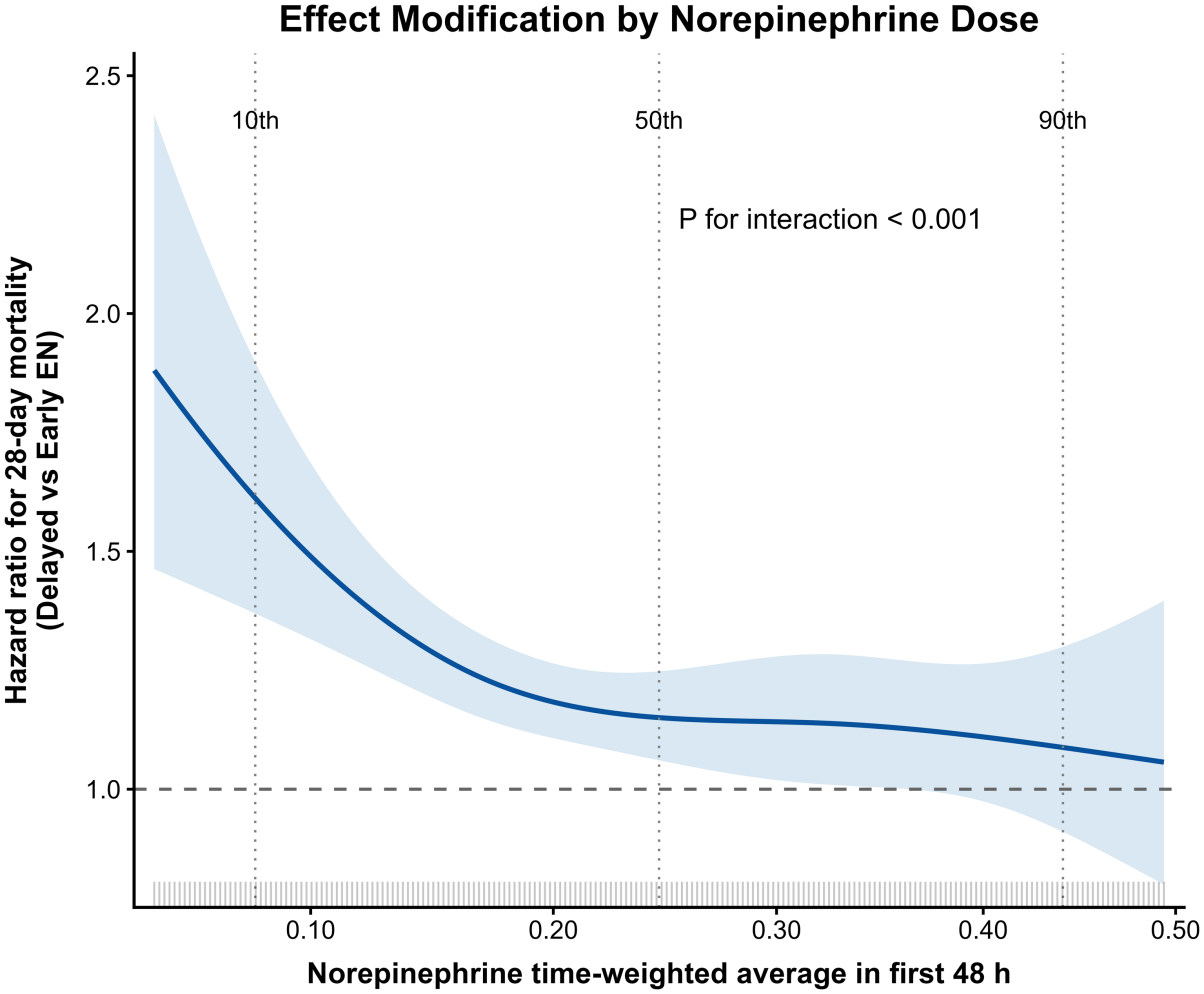
Interaction between norepinephrine dose and enteral nutrition timing for 28-day mortality. The curve shows the hazard ratio for delayed versus early EN, with shaded areas indicating the 95% confidence interval.

As shown in Figure 5, the association between EN timing and mortality varied according to norepinephrine exposure, with the hazard ratio comparing delayed versus early EN gradually decreasing as norepinephrine exposure increased. At higher levels of norepinephrine exposure, the difference between strategies approached unity. The interaction between norepinephrine exposure and nutrition timing was statistically significant (*p* for interaction <0.001).

### Sensitivity analyses

The sensitivity analyses were broadly consistent with the primary analysis. Using alternative definitions of the exposure window produced similar effect estimates (Supplementary Figure S3). Restricting the cohort using alternative operational definitions of septic shock also yielded comparable results (Supplementary Figure S4), supporting the robustness of the primary findings.

## Discussion

In this target trial emulation study of septic shock, delayed initiation of enteral nutrition was associated with higher 28-day mortality and fewer ventilator-free days compared with earlier initiation. The magnitude of this association appeared to vary with norepinephrine exposure, with the strongest difference observed at lower vasopressor intensity. Overall, hemodynamic status appears to be an important consideration when determining the timing of EN initiation in septic shock.

The observed interaction between EN timing and norepinephrine exposure is clinically plausible and consistent with previous reports. Patients with lower norepinephrine exposure may have more preserved splanchnic perfusion and therefore may be more likely to tolerate and potentially benefit from earlier EN. By contrast, as norepinephrine exposure increases, impaired gut perfusion and EN intolerance may become more relevant. At higher levels of norepinephrine exposure, the difference between early and delayed strategies was attenuated. However, this finding does not support a specific vasopressor threshold for clinical decision-making. The present analysis was intended to assess effect heterogeneity rather than to define a cutoff for safe enteral nutrition initiation. In patients with more severe hemodynamic instability, decisions regarding enteral nutrition should remain individualized and guided by overall perfusion status, vasopressor trajectory, and clinical tolerance rather than a fixed threshold. Similar patterns have been reported previously. Franzosi et al. (8) found that successful EN in septic shock was associated with more favorable perfusion parameters. Wang et al. (6) reported that indices incorporating arterial pressure and vasopressor dose were associated with EN timing in patients with shock. Other observational studies have likewise suggested that higher vasopressor exposure may be associated with lower EN tolerance or greater concern for gastrointestinal complications (9).

Beyond the observed interaction, our results also support an overall association between earlier EN initiation, lower mortality, and more ventilator-free days. This finding is consistent with previous studies examining nutritional strategies in septic shock and other critically ill populations. For example, in an observational cohort study of critically ill patients receiving EN, earlier initiation of EN was associated with improved survival and fewer complications (17). Similarly, in a multicenter cohort of mechanically ventilated patients with circulatory shock, early EN was associated with more favorable outcomes in unadjusted analyses, although this association attenuated after adjustment for illness severity (18). In addition, studies examining early nutritional support in critically ill populations have suggested that earlier nutrient delivery may reduce complications and improve recovery trajectories during critical illness (19). Taken together, these observations indicate that earlier EN may benefit selected patients, although the available evidence remains heterogeneous and inconclusive. Randomized evidence has also evaluated nutritional strategies in patients with circulatory shock, although results remain heterogeneous and have not consistently demonstrated mortality benefit (20). This interpretation is also consistent with prior review-based assessments of EN in circulatory shock and sepsis (10, 11).

This pattern is also biologically plausible. EN may help preserve gut barrier function and mucosal immunity during critical illness, thereby potentially reducing bacterial translocation and systemic inflammation, whereas impaired perfusion during shock may limit tolerance to EN, a mechanism consistent with the proposed role of intestinal barrier failure and bacterial translocation in the pathogenesis of multiple organ dysfunction during critical illness (21). In septic shock, the balance between potential benefit and risk is therefore likely to depend on the degree of hemodynamic instability. However, because detailed data on caloric and protein delivery, feeding progression, and cumulative energy balance were not available, the present study cannot fully elucidate the underlying nutritional mechanisms, and the observed associations should be interpreted primarily in terms of timing rather than the adequacy of nutritional support. In addition, outcomes such as infection complications, gastrointestinal intolerance, bowel ischemia, and parenteral nutrition use are not reliably captured as structured, time-resolved variables in the database, which limits their suitability for causal inference analyses in this setting. Length-of-stay outcomes also require cautious interpretation, as they may be influenced by competing risks such as early mortality and discharge practices.

Nevertheless, previous studies evaluating EN timing in septic shock have reported heterogeneous findings. Some investigations have suggested a benefit of early EN, whereas others have reported neutral results. For example, in a randomized pilot trial evaluating early trophic EN in septic shock, early EN was feasible but did not significantly improve mortality outcomes (22). Similarly, studies examining trophic EN strategies in septic shock have highlighted that nutritional interventions may produce different effects depending on illness severity and circulatory status (23). Observational analyses of nutritional interventions in critically ill populations have also suggested that the clinical impact of EN may vary across patient subgroups and clinical contexts (10). Differences in patient populations, definitions of early EN, and clinical practice patterns may therefore contribute to the variability observed across studies.

Differences among previous studies may also reflect the methodological challenges of evaluating treatment timing in observational data. In routine clinical practice, the decision to initiate EN evolves dynamically according to a patient’s clinical trajectory, including vasopressor dose, metabolic status, and overall hemodynamic stability. Conventional regression approaches that classify patients according to treatments received during follow-up may therefore introduce time-related biases when treatment initiation occurs after cohort entry, particularly immortal time bias and time-dependent confounding. Target trial emulation provides a framework for addressing these challenges by explicitly defining treatment strategies and aligning time zero with the start of follow-up, thereby enabling valid comparison of treatment strategies involving delayed EN. By emulating a target trial and applying a CCW framework, we aimed to reduce immortal time bias and time-dependent confounding and thereby provide a more robust evaluation of EN timing than conventional observational analyses. The use of discrete time windows in this study also enabled the comparison of clinically interpretable and actionable treatment strategies within the target trial framework.

This study has several strengths. First, the target trial emulation design allowed explicit specification of treatment strategies and follow-up time, helping to reduce common biases in observational studies of treatment timing. Second, the CCW analytic framework enabled evaluation of delayed treatment strategies while accounting for time-dependent treatment decisions and evolving clinical status. Third, the analysis was conducted using a large critical care database with detailed clinical information, allowing adjustment for a broad range of baseline characteristics and time-varying variables that may influence both nutritional decisions and clinical outcomes. These findings should be interpreted cautiously given the observational nature of the study.

### Limitations

Several limitations merit consideration. As with all observational studies, residual confounding cannot be completely excluded despite adjustment for measured variables. In addition, the analysis relied on routinely collected clinical data, and misclassification of exposures and covariates is possible.

In clinical practice, decisions regarding enteral nutrition initiation are influenced by dynamic and partially unmeasured factors, including clinician judgment, subtle changes in hemodynamic status, and bedside assessments that may not be fully captured in structured data. Although we adjusted for key baseline and time-varying variables, these may not fully reflect the complexity of real-time clinical decision-making. Therefore, the findings should be interpreted as associations under a causal inference framework rather than definitive causal effects.

This study was designed to evaluate the timing of enteral nutrition initiation rather than the adequacy or dose of nutritional delivery. Detailed information on caloric targets, protein provision, feeding progression, and cumulative energy balance was not consistently available in the database, making it difficult to distinguish between trophic feeding and full-dose enteral nutrition or to assess feeding adequacy. Accordingly, the findings should be interpreted in the context of initiation timing rather than as evidence on comprehensive nutritional strategies.

Another important limitation is the inability to assess gastrointestinal safety outcomes. Clinically relevant complications, such as feeding intolerance or bowel ischemia, are not reliably captured as structured, time-resolved variables in the database. In addition, diagnostic codes may underrepresent these conditions and do not provide sufficient temporal resolution to establish their relationship with enteral nutrition initiation. As a result, the safety profile of early enteral nutrition in septic shock remains uncertain in this analysis. Additional clinically relevant outcomes, including infection complications, gastrointestinal intolerance, bowel ischemia, and parenteral nutrition use, could not be robustly assessed due to limitations in structured, time-resolved data capture.

In addition, although early norepinephrine exposure was summarized using a time-weighted average dose, detailed vasopressor trajectories, duration above specific dose thresholds, and cumulative norepinephrine exposure were not separately modeled.

This study was based on data from a single tertiary academic center, and differences in patient populations, ICU organization, and nutrition practices across institutions may limit the generalizability of the findings. In addition, the study period spans more than a decade (2008–2019), during which sepsis management, hemodynamic resuscitation strategies, and enteral nutrition practices have evolved. These temporal changes may influence the applicability of the results to contemporary clinical settings. Therefore, the findings should be interpreted in the context of a real-world historical cohort, and further validation in more recent and multicenter populations is warranted.

## Conclusion

In this target trial emulation study of patients with septic shock, delayed initiation of EN was associated with higher mortality and fewer ventilator-free days compared with earlier initiation. Importantly, the magnitude of this association appeared to diminish as norepinephrine exposure increased, suggesting that the benefits of early EN may depend on the degree of circulatory instability. These findings indicate that the optimal timing of EN in septic shock may depend on the degree of hemodynamic instability.

## Data Availability

Publicly available datasets were analyzed in this study. The datasets analyzed in this study are publicly available. This data can be found: PhysioNet (https://physionet.org/), MIMIC-IV database.
