# Supplementary material for: Early versus delayed enteral nutrition in septic shock: a target trial emulation study
Source: Front Nutr. 2026 Jun 15;13:1831524. doi: 10.3389/fnut.2026.1831524 (PMC13310783; doi:10.3389/fnut.2026.1831524)
Supplement: Supplementary file 1 [file Table_1.docx]

**Supplementary Material**

**Title:**

Early Versus Delayed Enteral Nutrition in Septic Shock: A Target Trial Emulation Study

**Authors:**

Peng Zhang^1*^, Ying Huang^1*^, Xiangcheng Zhang^1*^, Xingxing Zhu^1^, Min Wang^1^, Hui Xia^2†^, Tongkun Zuo^1†^

**Affiliation:**

1. Department of Critical Care Medicine, The Affiliated Huai’an No. 1 People's Hospital of Nanjing Medical University, Huai’an, 223300, China
2. Department of Anesthesiology, The Affiliated Huaian No. 1 People’s Hospital of Nanjing Medical University, Huai’an 223300, China

**Corresponding author:**

Hui Xia

E-mail: xiahui0118@163.com

Tongkun Zuo

E-mail: yjqms@163.com

**Supplementary Tables**

| **Table S1. Specification of the hypothetical target trial and its emulation using observational data** | | |
| --- | --- | --- |
| **Component** | **Target trial** | **Emulation using observational data** |
| 1. Eligibility criteria | Adult ICU patients with septic shock and without contraindications to enteral nutrition | Adult ICU patients with septic shock identified from the MIMIC-IV database who met the operationalized eligibility criteria |
| 2. Treatment strategies | Early versus delayed initiation of enteral nutrition | Early enteral nutrition defined as initiation ≤48 hours after ICU admission; delayed enteral nutrition defined as initiation between 48 and 96 hours |
| 3. Assignment procedures | Random allocation to early or delayed enteral nutrition at ICU admission | Each eligible patient was cloned and assigned to both treatment strategies under a clone–censor–weighting framework |
| 4. Grace period | A treatment assignment window of 96 hours allowed to distinguish early vs delayed strategies | A 96-hour treatment assignment window was implemented to distinguish the two treatment strategies and reflect real-world clinical practice |
| 5. Start of follow-up (time zero) | Randomization at ICU admission | ICU admission defined as time zero |
| 6. End of follow-up | Death or 28 days after ICU admission | Death or 28 days after ICU admission |
| 7. Outcome | 28-day all-cause mortality | 28-day all-cause mortality |
| 8. Causal contrast | Per-protocol effect comparing strategy-adherent early vs delayed enteral nutrition | Per-protocol effect comparing strategy-adherent early vs delayed enteral nutrition |
| 9. Handling protocol deviations | Patients deviating from the assigned treatment strategy considered protocol violations | Clones were artificially censored when observed treatment no longer remained consistent with the assigned strategy, including initiation outside the assigned window or failure to initiate enteral nutrition within that window |
| 10. Confounding control | Randomization balances baseline confounders | Stabilized inverse probability weights used to adjust for baseline and time-varying confounders |
| 11. Weight construction | Not required | Stabilized inverse probability of censoring weights were estimated using pooled logistic regression models |
| 12. Positivity handling | Guaranteed by trial design | Stabilized weights were truncated at the 99th percentile to reduce the influence of extreme values |
| 13. Statistical analysis | Cox proportional hazards model comparing randomized groups | Weighted Cox proportional hazards model with robust standard errors clustered by stay identifier |
| 14. Sensitivity analyses | Pre-specified sensitivity analyses | Alternative exposure windows, alternative shock definitions, and complete-case analyses were conducted to assess robustness |
| ICU: Intensive care unit. | | |

| **Table S2. Observed distribution of enteral nutrition initiation** | | |
| --- | --- | --- |
| **A. Distribution of enteral nutrition initiation** | | |
| **EN initiation time** | **N** | **Percentage (%)** |
| ≤24 h | 177 | 4.4 |
| 24–48 h | 398 | 9.9 |
| 48–72 h | 402 | 10.0 |
| 72–96 h | 324 | 8.1 |
| No EN within 96 h | 2702 | 67.5 |

| **B. Summary statistics of enteral nutrition initiation time** | |
| --- | --- |
| **Statistic** | **Hours** |
| Median | 69.2 |
| Interquartile range (IQR) | 42.0–103.2 |

| **Table S3. Artificial censoring summary and diagnostics** | | |
| --- | --- | --- |
| **A. Artificial censoring rate** | | |
| **Strategy** | **Censored n** | **Percentage (%)** |
| Early | 3075 | 76.8 |
| Delayed | 2632 | 65.8 |

| **B. Censoring time among censored clones** | | |
| --- | --- | --- |
| **Strategy** | **Median censoring time (h)** | **Interquartile range (IQR), h** |
| Early | 48.0 | 48.0-48.0 |
| Delayed | 96.0 | 96.0-96.0 |

| **Table S4. Diagnostics of stabilized inverse probability of censoring weights** | |
| --- | --- |
| **Statistic** | **Value** |
| Mean weight | 1.040 |
| Median weight | 1.000 |
| 95th percentile | 1.229 |
| 99th percentile | 2.099 |
| Maximum weight | 19.934 |
| Effective sample size (ESS) | 78539 |
| ESS ratio | 0.823 |

| **Table S5. Definitions of baseline and time-varying covariates used in the target trial emulation** | | | | |
| --- | --- | --- | --- | --- |
| **Variable** | **Type** | **Operational definition** | **Time window** | **Role in analysis** |
| Age | Baseline | Age at ICU admission, in years | At ICU admission | Baseline confounder |
| Sex | Baseline | Recorded biological sex | At ICU admission | Baseline confounder |
| Race | Baseline | Categorized race/ethnicity variable derived from the admission record | At ICU admission | Baseline confounder |
| Admission type | Baseline | Admission category derived from the hospital admission record | At ICU admission | Baseline confounder |
| CCI | Baseline | Comorbidity burden quantified using the CCI | At ICU admission | Baseline confounder |
| APS III | Baseline | APS III at ICU admission | At ICU admission | Baseline confounder |
| SOFA score | Baseline | Sequential Organ Failure Assessment score at ICU admission | At ICU admission | Baseline confounder |
| Infection site | Baseline | Prespecified infection site category based on the primary infectious source | At ICU admission / baseline assessment | Baseline confounder |
| RRT within 96 h | Baseline / early course descriptor | Indicator of receipt of continuous renal replacement therapy within the first 96 h | First 96 h after ICU admission | Baseline descriptive variable / subgroup descriptor |
| EN initiation time | Exposure | Time from ICU admission to first enteral nutrition initiation | 0–96 h after ICU admission | Treatment strategy assignment / artificial censoring |
| Lactate measurement availability | Time-varying | Indicator of whether a lactate value was available in the prespecified look-back window | Updated every 6 h | Time-varying confounder in weight model |
| Most recent lactate value | Time-varying | Most recent prior lactate concentration carried forward within the allowable look-back window | Updated every 6 h | Time-varying confounder in weight model |
| Norepinephrine exposure indicator | Time-varying | Indicator of norepinephrine exposure during the prior interval | Updated every 6 h | Time-varying confounder in weight model |
| Norepinephrine dose | Time-varying | Lagged norepinephrine dose summary from the prior interval | Updated every 6 h | Time-varying confounder in weight model |
| Shock proxy | Time-varying cohort-defining variable | Operational septic shock indicator based on norepinephrine exposure together with recent lactate >2 mmol/L | Updated every 6 h during the first 96 h | Eligibility / cohort construction |
| Time since ICU admission | Time index | Discrete follow-up time represented in 6-hour intervals | Updated every 6 h | Included in numerator and denominator weight models |
| **Table S5 continued** | | | | |
| Time-weighted average norepinephrine dose | Derived exposure modifier | Time-weighted average norepinephrine dose during the first 48 h after ICU admission | 0-48 h after ICU admission | Effect heterogeneity analysis |
| 28-day mortality | Outcome | All-cause mortality within 28 days after ICU admission | Follow-up to day 28 | Primary outcome |
| Ventilator-free days at day 28 (VFD28) | Outcome | Number of days alive and free from invasive mechanical ventilation during the first 28 days; patients who died within 28 days were assigned zero | Follow-up to day 28 | Secondary outcome |
| CCI: Charlson comorbidity index; SOFA: Sequential Organ Failure Assessment Score; APS III: Acute Physiology Score III; ICU: Intensive care unit. | | | | |

| **Table S6. Weight model specification for stabilized inverse probability of censoring weights** | |
| --- | --- |
| **Model component** | **Covariates included** |
| Numerator model | Time since ICU admission (modeled over discrete 6-hour intervals) |
| Denominator model | Time since ICU admission; age; sex; race/ethnicity; admission type; CCI; APS III; SOFA score; lactate measurement availability indicator; most recent lactate value (lagged); norepinephrine exposure indicator; lagged norepinephrine dose; norepinephrine dose missingness indicator |
| Time-updated covariate handling | All time-varying covariates were incorporated in lagged form so that covariate values preceded the treatment decision and artificial censoring process in each interval |
| Weight construction | Stabilized inverse probability of censoring weights were calculated as the ratio of predicted probabilities from the numerator and denominator pooled logistic regression models |
| Weight truncation | Final stabilized weights were truncated at the 99th percentile to limit the influence of extreme weights |
| Weight application | Weights were estimated during the 0-96 h treatment assignment window and then carried forward unchanged for the remainder of follow-up in the weighted Cox model |
| CCI: Charlson comorbidity index; SOFA: Sequential Organ Failure Assessment Score; APS III: Acute Physiology Score III; ICU: Intensive care unit. | |

| **Table S7. Data sources of study variables in the MIMIC-IV database** | |
| --- | --- |
| **Variable** | **Data source** |
| Age | Provided in MIMIC-IV module “hosp” table “patients” |
| Sex | Provided in MIMIC-IV module “hosp” table “patients” |
| Race/ethnicity | Provided in MIMIC-IV module “hosp” table “admissions” |
| Admission type | Provided in MIMIC-IV module “hosp” table “admissions” |
| CCI | Provided in MIMIC-IV module “derived” table “charlson” |
| SOFA score | Provided in MIMIC-IV module “derived” table “sofa” |
| APS III | Provided in MIMIC-IV module “derived” table “apsiii” |
| Infection site | Derived from diagnosis codes in the MIMIC-IV module “hosp” table “diagnoses_icd” |
| RRT within 96 h | Provided in MIMIC-IV module “derived” table “rrt” |
| EN initiation time | Provided in MIMIC-IV module “ICU” table “inputevents” |
| Lactate variables | Provided in MIMIC-IV module “hosp” table “labevents” |
| Norepinephrine exposure | Provided in MIMIC-IV module “ICU” table “inputevents” |
| Norepinephrine dose | Provided in MIMIC-IV module “ICU” table “inputevents” |
| Shock proxy | Derived from vasopressor infusion records in “inputevents” and lactate measurements in “labevents” |
| Time since ICU admission | Derived analytic variable based on “icustays.intime” |
| Time-weighted average norepinephrine dose | Derived from norepinephrine infusion records in “inputevents” |
| 28-day mortality | Provided in MIMIC-IV module “hosp” table “patients” |
| Ventilator-free days | Derived from mechanical ventilation records and survival data in the MIMIC-IV database |
| CCI: Charlson comorbidity index; SOFA: Sequential Organ Failure Assessment Score; APS III: Acute Physiology Score III; RRT: Renal replacement therapy; ICU: Intensive care unit. | |

| **Table S8. Covariate balance before and after weighting** | | |
| --- | --- | --- |
| **Variable** | **SMD before weighting** | **SMD after weighting** |
| Age | 0.000 | 0.00002 |
| SOFA | 0.000 | 0.00001 |
| APS III | 0.000 | 0.00002 |
| CCI | 0.000 | 0.00001 |
| Sex | 0.000 | 0.00001 |
| Race | 0.000 | 0.00003 |
| Admission type | 0.000 | 0.00001 |
| CCI: Charlson comorbidity index; SOFA: Sequential Organ Failure Assessment Score; APS III: Acute Physiology Score III. | | |

**Supplementary Figures**


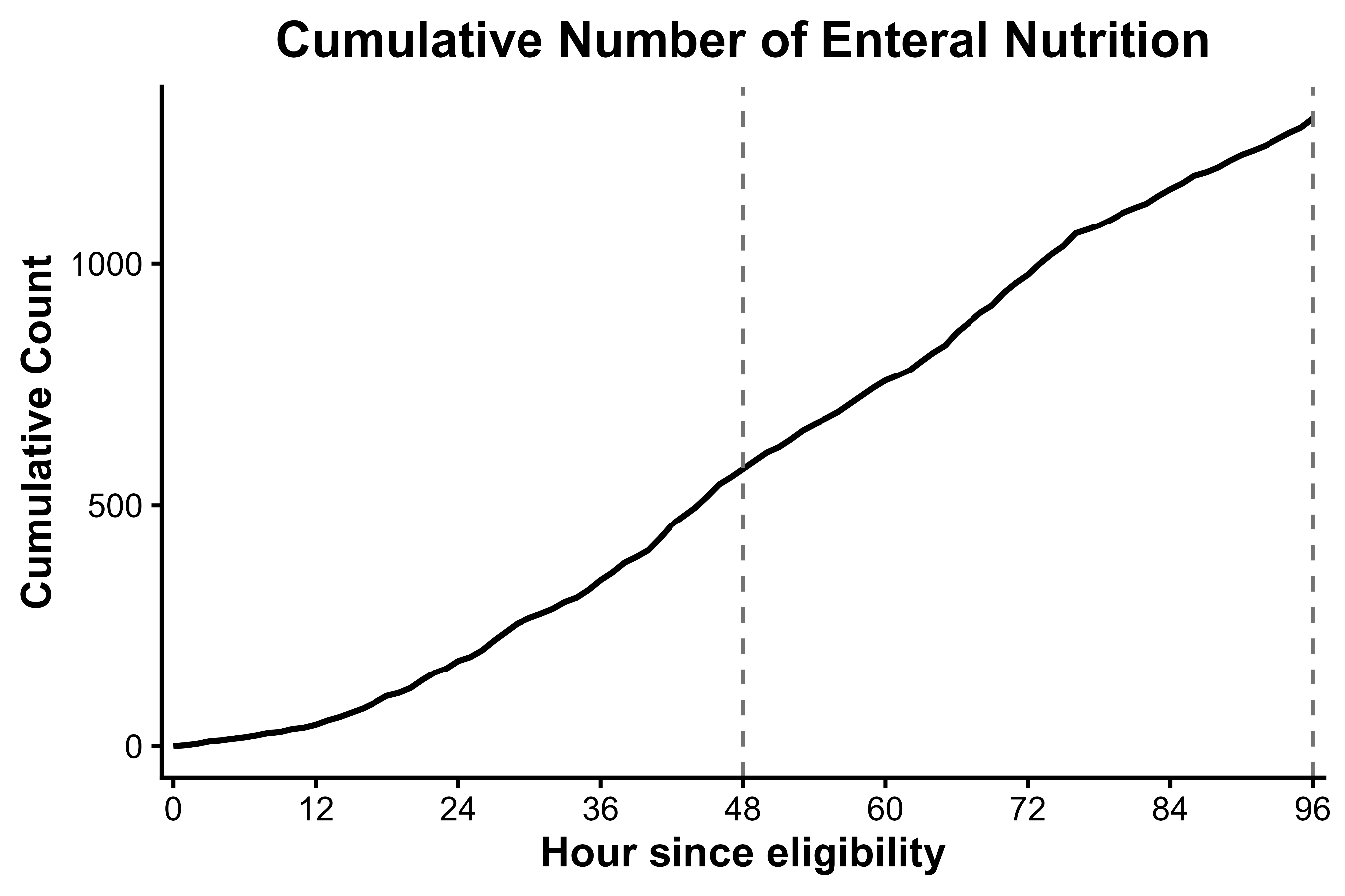


Fig S1. Cumulative number of enteral nutrition initiations over the study period. The moment of eligibility was defined as hour 0 and patients were followed for 96 h afterwards. The dashed vertical lines indicate the threshold for early enteral nutrition (48 h) and the end of the grace period (96 h).


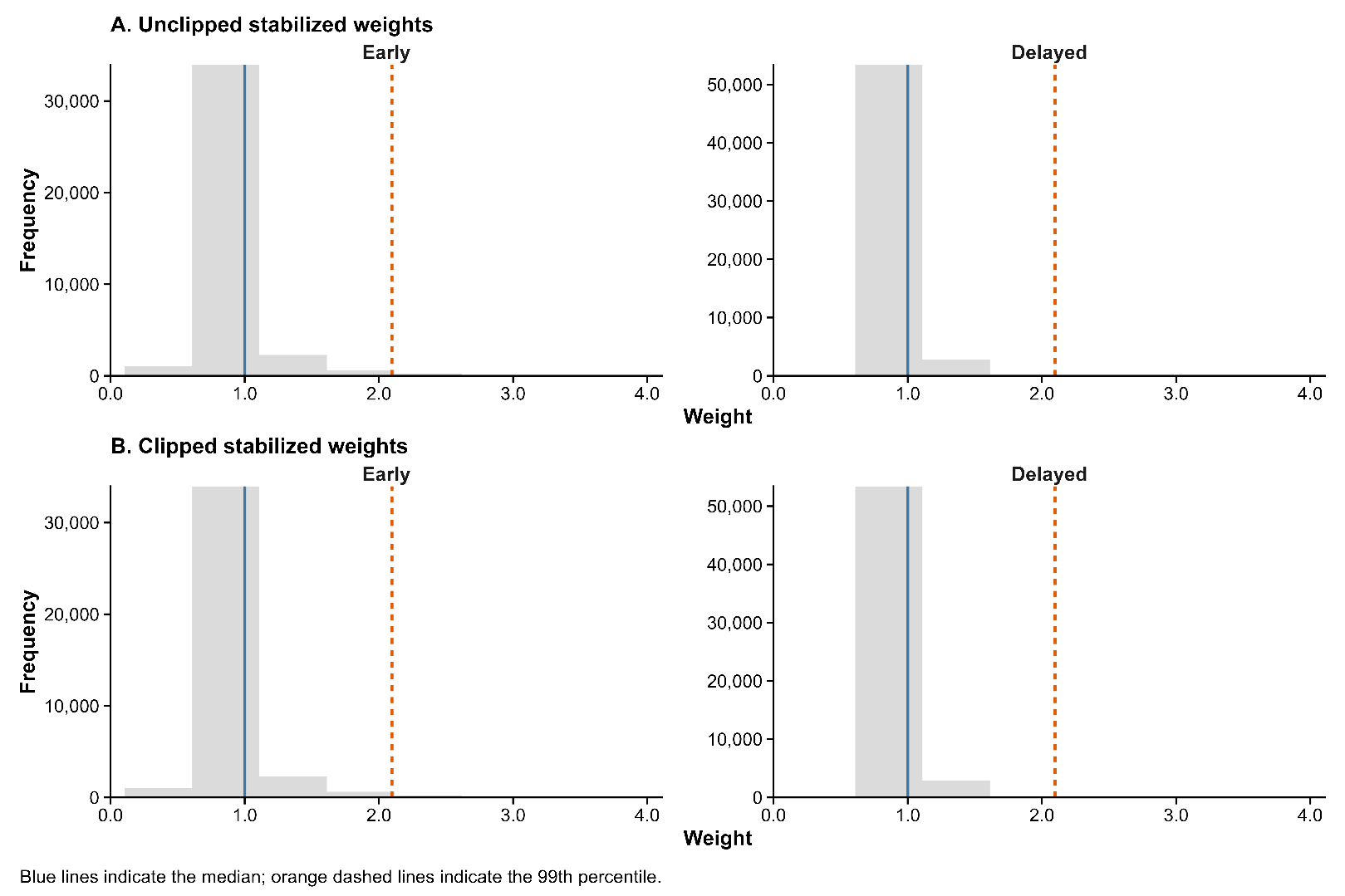


Fig S2. Histograms show the distribution of stabilized censoring weights for the early and delayed enteral nutrition strategies. Panel A shows the unclipped stabilized weights and panel B shows the clipped stabilized weights. Blue lines indicate the median and orange dashed lines indicate the 99th percentile.


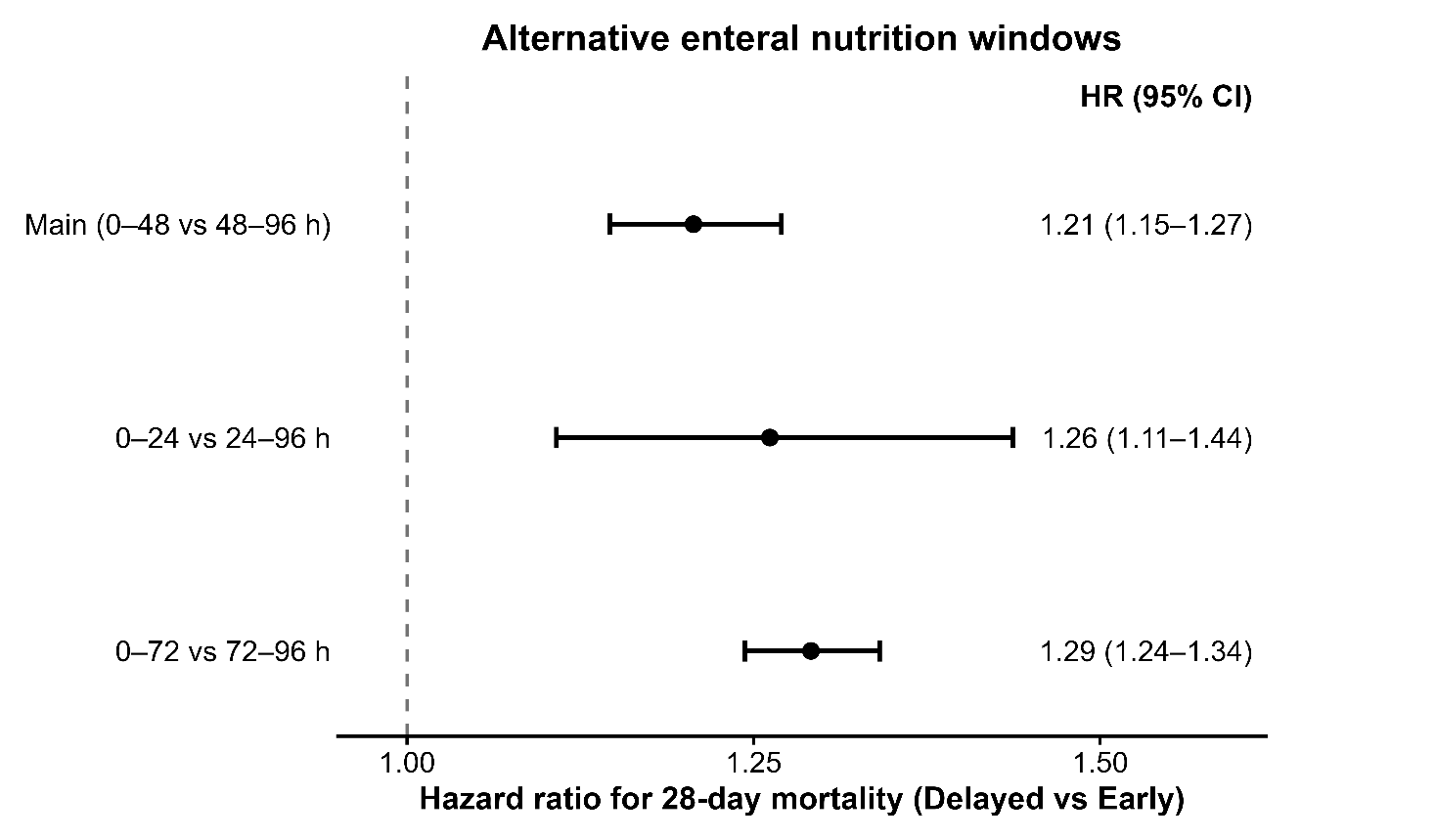


Fig S3. Sensitivity analysis using alternative exposure windows for enteral nutrition initiation. The association between delayed enteral nutrition and higher 28-day mortality remained consistent across alternative exposure windows.


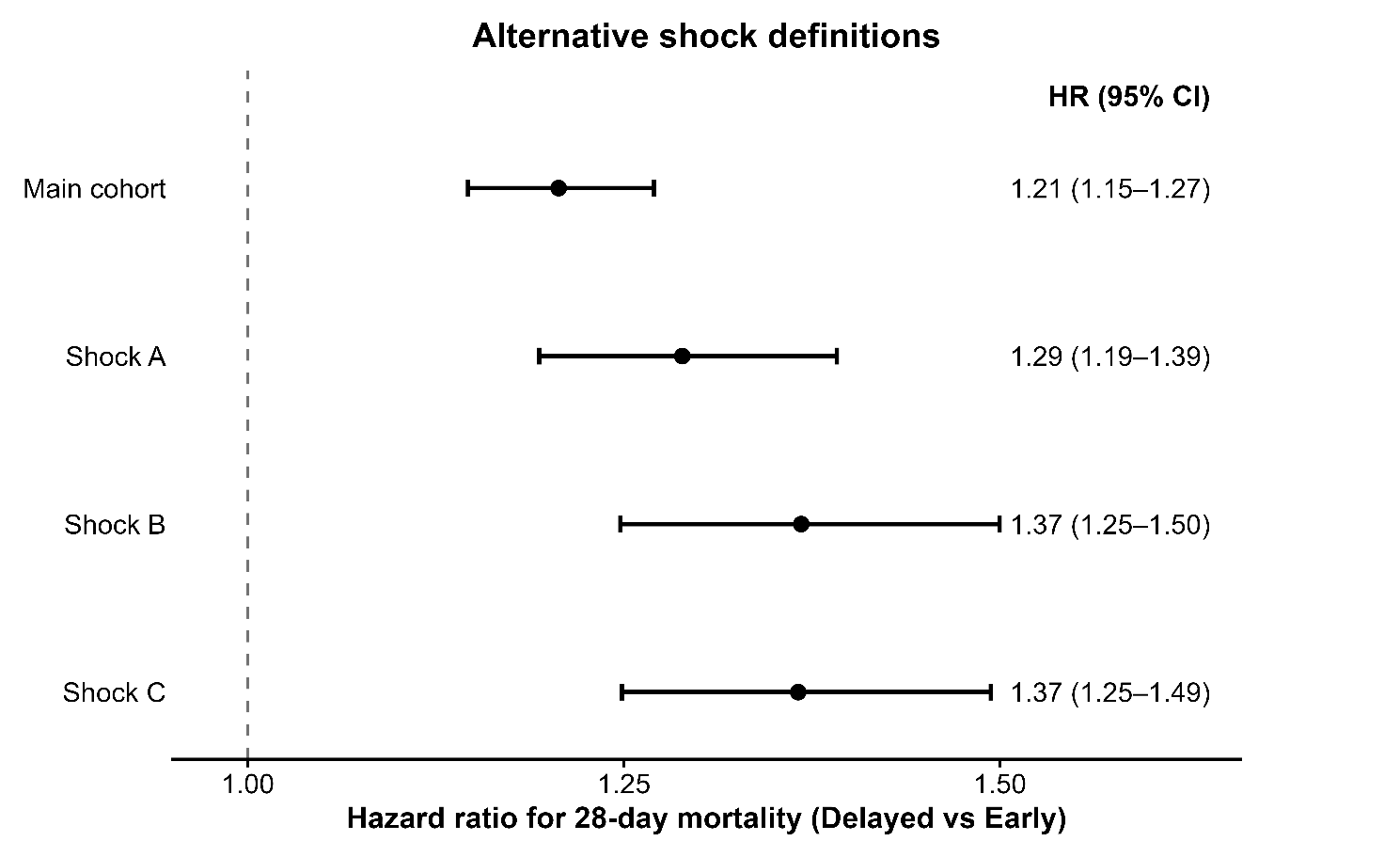


Fig S4. Sensitivity analysis using alternative definitions of septic shock. Hazard ratios compare delayed versus early enteral nutrition for 28-day mortality. Shock definitions were as follows: A, norepinephrine use within 24 h; B, lactate >2 mmol/L within 24 h; and C, norepinephrine use plus lactate >2 mmol/L within 24 h.

**Supplementary Checklist. STROBE Statement—Checklist of items that should be included in reports of observational studies**

STROBE Statement—Checklist of items that should be included in reports of ***cohort studies***

|  | Item No | Recommendation | Pages |
| --- | --- | --- | --- |
| **Title and abstract** | 1 | (*a*) Indicate the study’s design with a commonly used term in the title or the abstract | 1-5 |
|  |  | (*b*) Provide in the abstract an informative and balanced summary of what was done and what was found | 3-5 |
| Introduction | | |  |
| Background/rationale | 2 | Explain the scientific background and rationale for the investigation being reported | 6-8 |
| Objectives | 3 | State specific objectives, including any prespecified hypotheses | 8 |
| Methods | | |  |
| Study design | 4 | Present key elements of study design early in the paper | 8-9 |
| Setting | 5 | Describe the setting, locations, and relevant dates, including periods of recruitment, exposure, follow-up, and data collection | 8-9 |
| Participants | 6 | (*a*) Give the eligibility criteria, and the sources and methods of selection of participants. Describe methods of follow-up | 9 |
|  |  | (*b*) For matched studies, give matching criteria and number of exposed and unexposed | 9-10 |
| Variables | 7 | Clearly define all outcomes, exposures, predictors, potential confounders, and effect modifiers. Give diagnostic criteria, if applicable | 10-11 |
| Data sources/ measurement | 8* | For each variable of interest, give sources of data and details of methods of assessment (measurement). Describe comparability of assessment methods if there is more than one group | 11 |
| Bias | 9 | Describe any efforts to address potential sources of bias | 11-13 |
| Study size | 10 | Explain how the study size was arrived at | 9-10 |
| Quantitative variables | 11 | Explain how quantitative variables were handled in the analyses. If applicable, describe which groupings were chosen and why | 11 |
| Statistical methods | 12 | (*a*) Describe all statistical methods, including those used to control for confounding | 11-14 |
|  |  | (*b*) Describe any methods used to examine subgroups and interactions | 13-14 |
|  |  | (*c*) Explain how missing data were addressed | 11 |
|  |  | (*d*) If applicable, explain how loss to follow-up was addressed | NA |
|  |  | (*e*) Describe any sensitivity analyses | 13 |
| Results | | |  |
| Participants | 13* | (a) Report numbers of individuals at each stage of study—eg numbers potentially eligible, examined for eligibility, confirmed eligible, included in the study, completing follow-up, and analysed | 15-16 |
|  |  | (b) Give reasons for non-participation at each stage | 15 |
|  |  | (c) Consider use of a flow diagram | 15 |
| Descriptive data | 14* | (a) Give characteristics of study participants (eg demographic, clinical, social) and information on exposures and potential confounders | 15-16 |
|  |  | (b) Indicate number of participants with missing data for each variable of interest | 15-16 |
|  |  | (c) Summarise follow-up time (eg, average and total amount) | 15-16 |
| Outcome data | 15* | Report numbers of outcome events or summary measures over time | 16-17 |
| Main results | 16 | (*a*) Give unadjusted estimates and, if applicable, confounder-adjusted estimates and their precision (eg, 95% confidence interval). Make clear which confounders were adjusted for and why they were included | 17-18 |
|  |  | (*b*) Report category boundaries when continuous variables were categorized | NA |
|  |  | (*c*) If relevant, consider translating estimates of relative risk into absolute risk for a meaningful time period | 16 |
| Other analyses | 17 | Report other analyses done—eg analyses of subgroups and interactions, and sensitivity analyses | 17-18 |
| Discussion | | |  |
| Key results | 18 | Summarise key results with reference to study objectives | 18 |
| Limitations | 19 | Discuss limitations of the study, taking into account sources of potential bias or imprecision. Discuss both direction and magnitude of any potential bias | 22-23 |
| Interpretation | 20 | Give a cautious overall interpretation of results considering objectives, limitations, multiplicity of analyses, results from similar studies, and other relevant evidence | 18-23 |
| Generalisability | 21 | Discuss the generalisability (external validity) of the study results | 22-23 |
| Other information | | |  |
| Funding | 22 | Give the source of funding and the role of the funders for the present study and, if applicable, for the original study on which the present article is based | 24 |

*Give information separately for exposed and unexposed groups.

**Note:** An Explanation and Elaboration article discusses each checklist item and gives methodological background and published examples of transparent reporting. The STROBE checklist is best used in conjunction with this article (freely available on the Web sites of PLoS Medicine at http://www.plosmedicine.org/, Annals of Internal Medicine at http://www.annals.org/, and Epidemiology at http://www.epidem.com/). Information on the STROBE Initiative is available at http://www.strobe-statement.org.
